# Ginsenoside Re Attenuates High Glucose-Induced RF/6A Injury *via* Regulating PI3K/AKT Inhibited HIF-1α/VEGF Signaling Pathway

**DOI:** 10.3389/fphar.2020.00695

**Published:** 2020-05-21

**Authors:** Weijie Xie, Ping Zhou, Muwen Qu, Ziru Dai, Xuelian Zhang, Chenyang Zhang, Xi Dong, Guibo Sun, Xiaobo Sun

**Affiliations:** ^1^Institute of Medicinal Plant Development, Peking Union Medical College and Chinese Academy of Medical Sciences, Beijing, China; ^2^Guang'anmen Hospital, Chinese Academy of Chinese Medical Sciences, Beijing, China

**Keywords:** ginsenoside Re, diabetic retinopathy, oxidative stress, apoptosis, phosphoinositide 3-kinase/AKTT, hypoxia-inducible factor-1-alpha, vascular endothelial growth factor

## Abstract

Hyperglycaemia-induced retinal microvascular endothelial cell apoptosis is a critical and principle event in diabetic retinopathy (DR), which involves a series of complex processes such as mitochondrial dysfunction and oxidative stress. Ginsenoside Re (Re), a key ingredients of ginseng, is considered to have various pharmacologic functions, such as antioxidative, inhibition of inflammation and anti-apoptotic properties. However, the effects of Re in DR and the related mechanisms of endothelial cell injury induced by high glucose (HG) exposure remain unclear. The present study was designed to investigate and evaluate the ability of Re to ameliorate HG-induced retinal endothelial RF/6A cell injury and the potential mechanisms involved in the hypoxia-inducible factor-1-alpha (HIF-1α)/vascular endothelial growth factor (VEGF) signaling regulated by phosphoinositide 3-kinase (PI3K)/AKT pathway. Our results showed that preincubation with Re exerted cytoprotective effects by reversing the HG-induced decrease in RF/6A cell viability, downregulation of apoptosis rate and inhibition of oxidative-related enzymes, thereby reducing the excess intracellular reactive oxygen species (ROS) and HG-triggered RF/6A cell injury. In addition, Western blot analysis results showed ginsenoside Re significantly increased HIF-1α expression in the cytoplasm but decreased its expression in the nucleus, suggesting that it reduced the translocation of HIF-1α from the cytoplasm to the nucleus, and downregulated VEGF level. Moreover, this effect is involved in the activation of the PI3K/Akt pathway. LY294002, a PI3K inhibitor, was used to block the Akt pathway. Afterwards, the effects of Re on the regulation of apoptotic related proteins, VEGF and HIF-1α nuclear transcription was partially reversed. These findings suggested the exerting protective effects of ginsenoside Re were associated with regulating of PI3K/AKT and HIF-1α/VEGF signaling pathway, which indicates that ginsenoside Re may ameliorates HG-induced retinal angiogenesis and suggests the potential for the development of Re as a therapeutic for DR.

## Introduction

Diabetes mellitus (DM), a metabolic disease that mainly manifested as hyperglycemia, causes series of diabetes‐related vascular complications, such as diabetic encephalopathy, diabetic nephropathy, and diabetic retinopathy (DR) (Beckman and Creager, 2016; Zheng et al., 2018). DR, a common and severe microvascular complication of DM, is believed to be the main cause of blindness among working-age individuals worldwide (Keech et al., 2007). This long-term pathological process is triggered by hyperglycaemia during the development of diabetes and generally defined as two stages according to severity, non-proliferative DR (NPDR) and proliferative DR (PDR), which results in vision loss and reduce the quality of patients' life significantly (Aiello, 2014). Among a series of complex mechanisms involved in DR, oxidative stress induced by chronic hyperglycaemia has been identified as the principal pathogenic factor in various cell types (Kowluru and Chan, 2007).

These chronic complications of diabetes are important causes of death and disability, creating in a major public health burden. DR is defined as a serious microvascular complication in patients suffering from DM and the primary cause of blindness in working-age people of developed countries (Alswailmi, 2018; Cui et al., 2018; Liu et al., 2018). At present, the mechanisms leading to DR are not fully understood, but the common opinions insist that vascular endothelial cell migration and microvascular proliferation caused by vascular endothelial growth factor (VEGF) overexpression may be some of the most important mechanisms underlying the development of DR (Mazidi et al., 2017; Olivares et al., 2017; Shi et al., 2018). Hypoxia-inducible factor-1-alpha (HIF-1α), a major regulator of VEGF transcription, has been shown to be closely associated with hyperglycaemia and insulin secretion (Cui et al., 2018; Liu et al., 2018). In addition, increased reactive oxygen species (ROS) overexpression are frequently detected in the retinas of diabetic patients, and these levels can be improved by antioxidants. Excess ROS can result in the release of VEGF, a predominant factor that promotes neovascularization, leading to further stimulation of the inflammatory response (Zheng et al., 2009). Moreover, accumulating evidence has suggested that HIF-1α, a factor vital for hypoxic adaptation that interacts with ROS and the VEGF pathway, is also involved in endothelial dysfunction and apoptosis (Palmer et al., 1998; Pore et al., 2006; Jing et al., 2012). Hypoxia activates a range of target genes including HIF-1α which can further regulate of VEGF transcription, that considered to be the most important intraocular neovascularization factor (Wang et al., 2014). These reports suggest that HIF-1α/VEGF pathway is crucial for facilitating the process of DR, and diabetes-related injury are reduced after its genes are interfered with, by mechanisms which are related to Akt activation (Jo et al., 2014).

Thus, the amelioration of high glucose (HG)-triggered endothelial cell oxidative and apoptosis is a potential target for protecting against DR. Hence, it is vital to discover and apply new natural active ingredients targeting the HIF-1α/VEGF signal pathway that exert marked effects on the retina in patients with DM.

**Figure 1:**
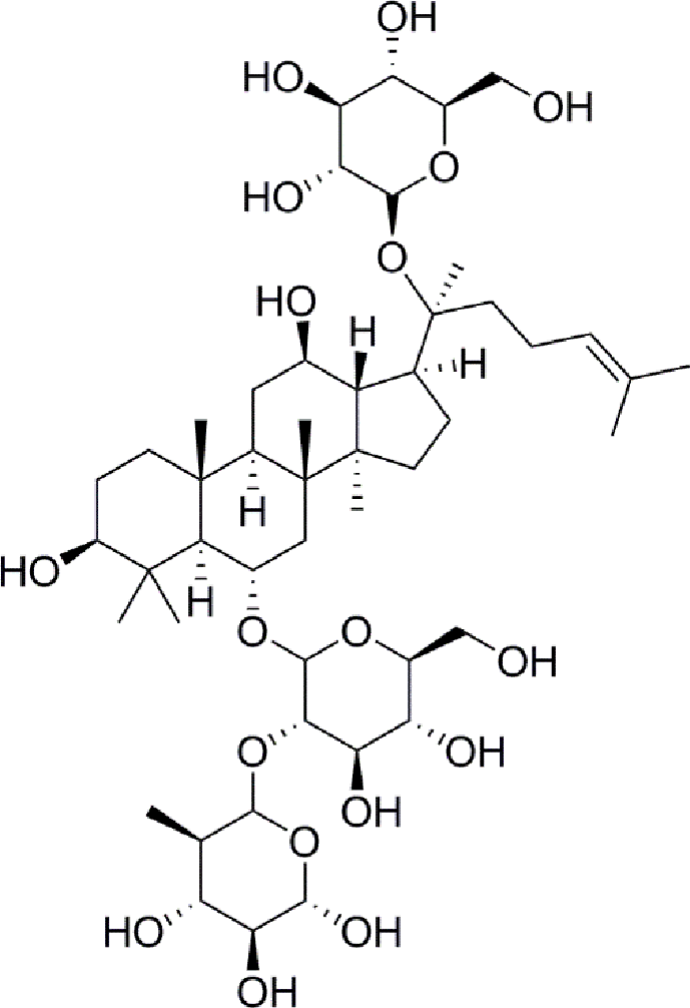
Chemical structure.

Panax notoginseng has a long history as a botanical drug in Asia and is used to treat diseases. Panax notoginseng saponins (PNS) are the most abundant extracts of the roots of Panax notoginseng and have long been used to treat diabetes (Zhang et al., 2016; Fan et al., 2017). Such as, notoginsenoside Ft1 was reported to enhance platelet aggregation through P2Y12 (Zhang et al., 2016), and notoginsenoside R1 inhibites HG-caused endothelial injury by regulating the oxidative stress process (Fan et al., 2017). Ginsenoside Re (**Figure 1**) is a protopanaxatriol-type ginsenoside extracted from Panax *notoginseng* and Panax *ginseng* (Xie et al., 2018). ginsenoside Re has multiple biological activities, including antidiabetes, antioxidative, anti-inflammatory, and antitumor effects (Meng et al., 2018). Moreover, a new evidence has shown that ginsenoside Re relieves hyperglycemia and hyperlipidemia in the diabetes model (Xie et al., 2005), and it regulates the redox state in streptozotocin-induced diabetic rats (Cho et al., 2006). Whareas, the function and the mechanisms of ginsenoside Re against diabetes-induced retinal injury remain unclear, and the mechanisms have not been determined *via* the HIF-1α/VEGF signal pathway.

Furthermore, the activited phosphoinositide 3-kinase (PI3K)/Akt signaling pathway, which is critical for maintaining retinal cell function, can protect from the HG-induced retinal damage (Jacot and David, 2011). A recent study has shown that Re exerts its antioxidative effects through the PI3K/Akt signaling pathway (Nakaya et al., 2007).

According to these evidences, we hypothesized that ginsenoside Re may protect against HG-induced RF/6A cells injury *via* the PI3K/AKT regulated HIF-1α/VEGF signal pathway. Hence, this study was performed to explore the effects and mechanisms of ginsenoside Re against DR by HG-induced retinal vascular injury model. Firstly, our results indicate that Re can ameliorate the oxidative response and apoptotic injury in RF/6A cells and that the protective potential mechanism of ginsenoside Re may regulate PI3K/AKT and HIF-1α/VEGF pathway inhibition.

## Methods

### Cell Culture

The monkey retinal vascular endothelial RF/6A cells were obtained from American Type Culture Collection (ATCC). Cells were propagated in RPMI1640 medium supplemented with 10% fetal bovine serum (FBS) at 37°C in the cell incubator with 5% CO_2_ and 95% air. The stock solution of ginsenoside Re (1 M) was preserved in dimethyl sulfoxide (DMSO) and diluted to different concentrations in serum-free medium before use. HG (50 mM) and LY294002 (50 μmol/L for 2 h) was prepared in serum-free medium immediately before incubation. The experimental design was shown in the **Supplementary Material Table 2**.

### MTT Assay

The survival rate of RF/6A cells was detected with MTT assay. RF/6A cells were planted on a 96-well plate (1 × 10^5^ cells/well). RF/6A cells were preincubation with ginsenoside Re as required, After rinsing with phosphate-buffered saline (PBS), the medium containing corresponding concentration of glucose was used to incubate sequentially. Afterwards, MTT was diluted to 1 mg/ml and then replaced in the plate followed by incubating at 37°C for 4 h. Next, 100 μl of DMSO was supplemented into each well. After shaking for 60s, the absorbance was detected at 560 nm.

### Determination of ROS

Intracellular and mitrochoindrial ROS level was detected using a fluorescent probe DCFH-DA and an Image-iT LIVE Green ROS Detection Kit (Invitrogen, CA, USA). RF/6A cells were planted in 6-well plates (1 × 10^5^ cells/well), rinsed with PBS, followed by treating with 10 μM DCFH-DA for 20 min at cell incubator. Mitochondrial ROS levels were determined with flow cytometry (BD Biosciences, USA).

### Detection of Catalase, Malondialdehyde, Glutathione Peroxidase, and Lactate Dehydrogenase

The levels of redox markers, including catalase (CAT), malondialdehyde (MDA), glutathione peroxidase (GSH-Px), and lactate dehydrogenase (LDH), were evaluated with corresponding assay kits purchased from Nanjing Jiancheng Bioengineering Institute. RF/6A cells (1 × 10^5^ cells/ml) were seeded in six-well plates. 3 μM Re was used to incubate the cells for 24 h, and then replaced with 50 mM HG. LDH release was detected using cell supernatant. And then intracellular LDH, MDA, CAT, and GSHPX activities were detected by cell disruption. LDH production was calculated with the rate of extracellular LDH to total LDH. The level of each oxidative stress indicator is presented as a percent of the control.

### Detection of ΔΨm

ΔΨm was detected by JC-1 (Enzo Life Sciences International, USA) fluorescent dye labeling. In each group, RF/6A cells (1 × 10^5^ cells/ml) seeded on 12-well plates were pretreated using ginsenoside Re (3 μM) for 24 h and then treated by HG (50 mM). Then the JC-1 working solution was used to treat cells cell incubator. The stained cells were rinsed twice with PBS followed by being photographed with a fluorescence microscope (Molecular Devices, USA). The Image J software was used to analyze the intensity of the fluorescence.

### Quantification of the Apoptosis Rate

Annexin V-PI experiment was performed to quantify the ratio of apoptotic cells with flow cytometry. RF/6A cells (1 × 10^5^ cells/well) were planted in six-well plates. 3 μM ginsenoside Re was used to treat cells for 24 h, after being rinsed with PBS, cells were treated with HG (50 mM) for 24 h. Then, the cells were treated with 100 μl of binding buffer supplemented with 5 μl annexin V and PI (1 μg/ml) for 15 min. 400 μl binding buffer was added followed by detecting with a FACSCalibur analysis (BD Biosciences, USA).

### Detection of DNA Fragmentation

To quantify the proportion of DNA fragmentation in different groups of cells, Cells were cultured in six-well plates. After different treatments, 4% paraformaldehyde solution was used to fix the cells. Next, the cells were incubated with 0.1% Triton X-100 for 10 min, and then rinsed in the washing solution. The cells were incubated in the cell incubator with the terminal deoxynucleotidyl transferase in the kit for 1 h, rinsed with PBS, and treated with the configured anti-digoxigenin for half an hour. After being washed with PBS, RF/6A cells were incubated using DAPI. The pictures were obtained with the fluorescence microscope.

### Western Blot Analysis

After extracting proteins and determining their concentration (unified to be 5 μg/μl), proteins of the same volume and concentration were added to the wells of the precast gel, then transferred to a membrane, as previously reported (Zhou et al., 2017). Next, Then the blocking solution was used to block the membrane for at least 2 h, following the primary antibodies against HIF-1α (ab203848; 1:2000), cleaved caspase-3 (ab32042; 1:500), cleaved caspase-9 (ab2324; 1:1000), VEGF ((sc-7269; 1:500), and lamin B (ab133741; 1:2000) and secondary antibodies were used to incubate with membranes. The membranes were washed by Tris Buffered saline Tween (TBST) for three times and then observed by the Molecular Imager System.

### Statistical Analysis

Results are expressed as the mean ± standard error of the mean. The data of different groups were compared using Student's t-test or ANOVA by Prism 5.00. P value < 0.05 was considered significant.

## Results

### Ginsenoside Re Preconditioning Improved Cell Viability Against HG-Induced RF/6A Cell Injury

**Figure 2:**
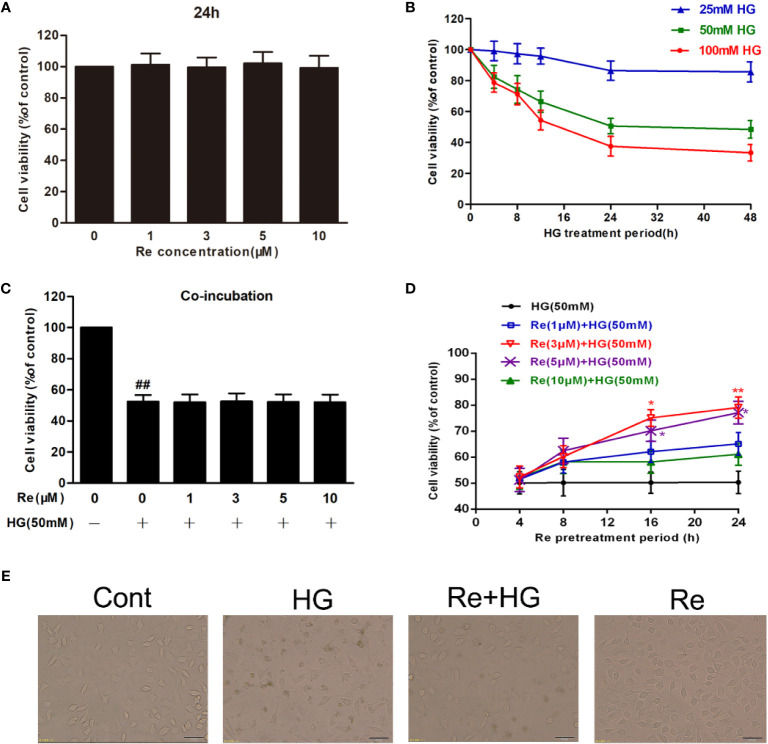
Ginsenoside Re protects RF/6A cells against HG-induced injury. **(A)** The effect of HG on RF/6A cells at different concentrations for various times. **(B)** The coincubation with Re and HG for 24 h. **(C)**, The pretreatment with Re for 24 h, and incubation by HG for 24 h. **(D)** The toxic effect of Re treatment on RF/6A cells. **(E)** The pretreatment of Re (3 μM) for 24 h and the incubation with HG (50 mM) for another 24 h. Cell activity was tested by MTT. Morphological observation was conducted by an inverted microscope. The data are presented as the mean ± standard error of the mean (n = 5). ^##^*P* < 0.01 versus the control group; *P < 0.05, **P < 0.01 versus the HG group. Scale bar, 100 μm. HG, high glucose.

Based on related literature (Yan et al., 2015; Du et al., 2017), the potential toxic and injury effects of HG and Re on RF/6A cells were estimated by MTT detection. As demonstrated in **Figure 2A**, after the cells were pretreated with Re at a serious doses (0, 1, 3, 5, and 10 μM) for 24 h, no significance difference was revealed (*P* > 0.05). In contrast, the cell activity of RF/6A cells significantly decreased with increasing HG concentration (25, 50, and 100 mM) in a time-dependent manner (4, 8, 12, and 24 h; shown in **Figure 2B**). Treatment of RF/6A cells with HG (50 mM) for 24 h results in the 50% of cell viability reduction (*P* < 0.01, **Supplementary Material Table 1**). Thus, 50 mM HG and a 24-h treatment period was used in the following experiment.

When the cells were preincubated with a serious doses of Re (0, 1, 3, 5, and 10 μM) for 24 h followed by 50 mM of HG treatment, cell viability changed markedly, and 3 μM of Re demonstrated a significant cytoprotective effect (**Figure 2D**). However, almost no protection was observed when Re at any concentration tested (0, 1, 3, 5, and 10 μM) was coincubated with HG for 24 h (**Figure 2C**, *P* > 0.05), which indicates that the protective effect of Re occurred merely in the context of preconditioning. Subsequently, the cytotoxic effect of Re was measured, the results indicates no significant difference (**Figure 2A**, *P* > 0.05). Moreover, morphological images showed that Re obviously reversed the cell shrinkage caused by HG induction, irregular shape, and opaque texture, etc. (**Figure 2E**), which was consistent with the above results. Thus, 3 μM ginsenoside Re was used in subsequent experiments.

### Ginsenoside Re Suppressed HG-Induced Oxidation

**Figure 3:**
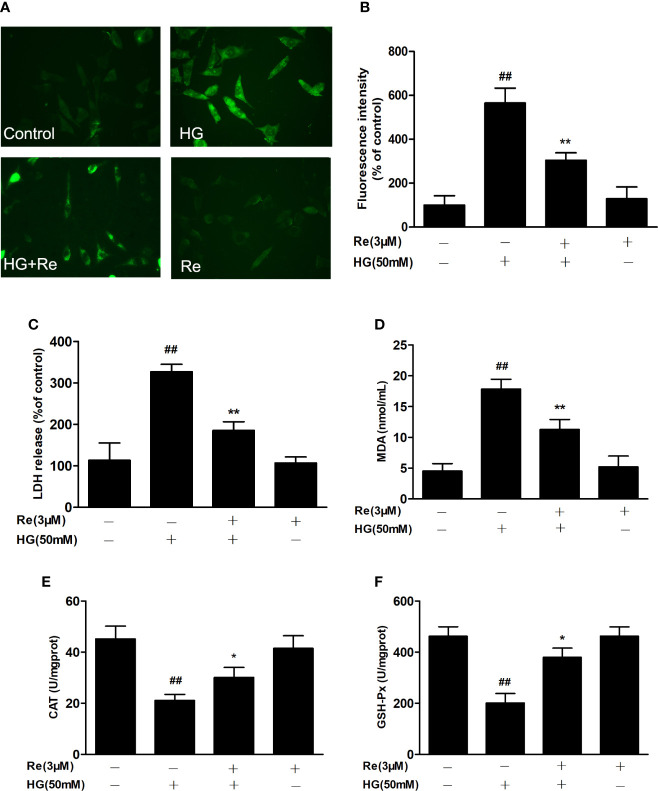
Ginsenoside Re attenuated HG-induced RF/6A cell injury and oxidative stress. **(A)** ROS levels were monitored using a fluorescence microscope. **(B)** Statistical analysis of ROS fluorescence intensity. The enzymatic activities of LDH **(C)**, MDA **(D)**, CAT **(E)**, and GSH-Px **(F)** were detected by spectrophotometry. The data are presented as the mean ± standard error of the mean (n = 5). ^##^*P* < 0.01 versus the control group; *P < 0.05, **P < 0.01 versus the HG group. Scale bar, 50 μm. ROS, reactive oxygen species; LDH, lactate dehydrogenase; MDA, malondialdehyde; CAT, catalase; GSH-Px, glutathione peroxidase.

To further assess the effects of ginsenoside Re on hyperglycaemia-induced RF/6A cell injury, the intracellular level of ROS, the enzymatic activities of LDH, MDA, CAT, and GSH-Px were detected. As exhibited in **Figure 3**, the production of intracellular ROS, LDH, and MDA were significantly upregulated in the HG-treated group (**Figures 3A, B**, *P* < 0.01; **Figure 3C**, *P* < 0.01 and **Figure 3C**, *P* < 0.01), indicating that HG exerts its cytotoxicity and injury. However, the pretreatment of Re evidently reduce the production of ROS (**Figure 3B**, *P* < 0.01), LDH (**Figure 3C**, *P* < 0.01) and MDA (**Figure 3D**, *P* < 0.01), indicating that significantly lessened HG-induced RF/6A cell cytotoxicity and injury. Furthermore, our reaches results revealed that the enzymatic activities of CAT and GSH-Px were markedly downregulated in HG group (**Figure 3E**, *P* < 0.01; **Figure 3F**, *P* < 0.01). Pretreatment with Re effectively increased the activities of CAT (**Figure 3E**, *P* < 0.05) and GSH-Px (**Figure 3F**, *P* < 0.05) in the HG-treated RF/6A cells. On the contrary, no significant differences were shown with Re incubation alone. The above data demonstrate that ginsenoside Re may protect against HG-triggered RF/6A cell injury and enhance anti-oxidative activity.

### Ginsenoside Re Improved Mitochondrial Function

**Figure 4:**
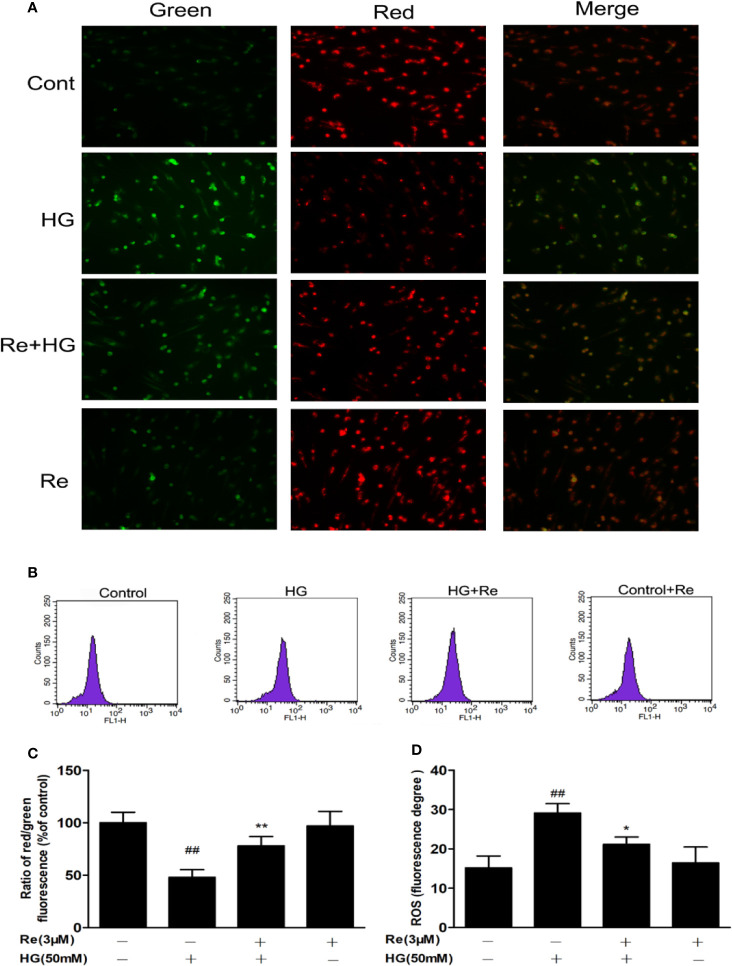
Effects of ginsenoside Re on ΔΨm in HG-treated RF/6A cells. **(A)** Ginsenoside Re inhibited the HG-triggered reduction in ΔΨm. **(B)** Mitochondrial ROS production in RF/6A cells was detected with flow cytometry. **(C)** The ratio of red fluorescence to green fluorescence. **(D)** Analysis of ROS fluorescence intensity. The results are presented as the mean ± standard error of the mean (n = 5). ^##^*P* < 0.01 versus the control group; *P < 0.05, **P < 0.01 versus the HG group. Scale bar, 50 μm.

The effect of HG on oxidative respiration is so severe and rapid that it directly destroys mitochondrial function and makes the mitochondrial membrane potential unbalanced, which in turn results in the production of apoptotic factors (Wang et al., 2018). In our study, ΔΨm was declined in HG-treated cells, as indicated by the results of JC-1 staining with flow cytometry. As demonstrated in **Figures 4A, C**, ginsenoside Re substantially protected against the ΔΨm loss caused by 50 μM glucose (*P* < 0.01). Conversely, the pretreatment of Re inhibited the decrease in ΔΨm in the mitochondria in HG-induced RF/6A cells (**Figure 4C**, *P* < 0.01). Moreover, mitochondrial ROS levels were evaluated to assess mitochondrial function. As shown in **Figures 4B, D**, there was an obviously increasing tendency in ROS generation in RF/6A cells at 24 h after HG treatment (*P* < 0.01), and this increase was ameliorated by the Re pretreatment (*P* < 0.05). The data indicated that the protective effect of Re involved reducing ROS production and improving mitochondrial function.

### Ginsenoside Re Attenuated the HG-Triggered Apoptosis in RF/6A Cells

**Figure 5:**
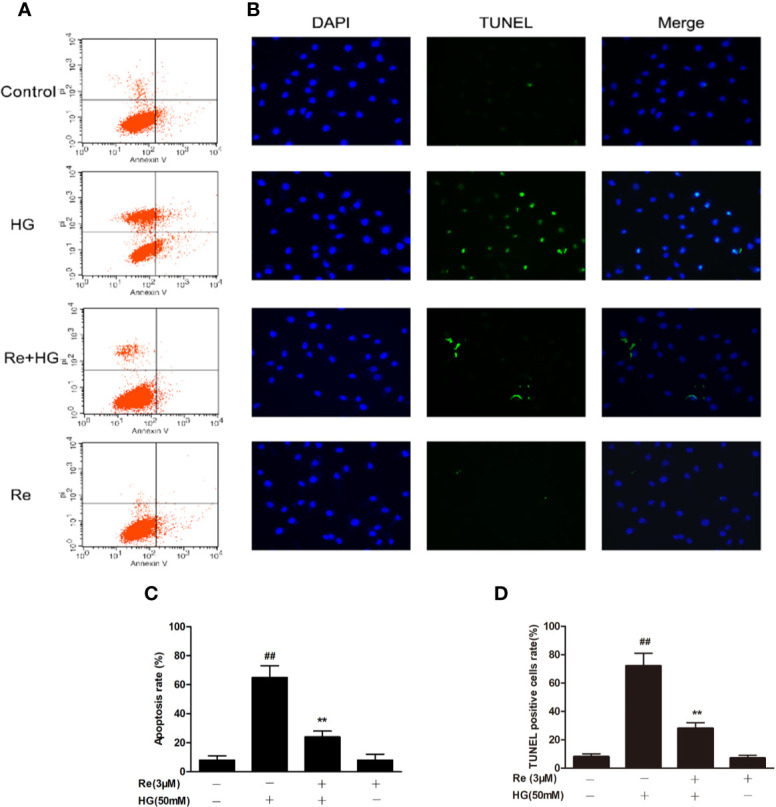
Effects of Ginsenoside Re on HG-triggered apoptosis in RF/6A cells. **(A)** Distribution map of apoptotic cells detected by annexin V/PI double staining. **(B)** Representative images captured with fluorescence microscopy showing TUNEL-stained RF/6A cells. **(C)** Quantitative analysis of the ratio of annexin V/PI-positive cells to total cells. **(D)** The ratio of TUNEL-positive cells. The results are expressed as the mean ± SE of the mean (n = 5). ^##^*P* < 0.01 versus the control group; **P < 0.01 versus the HG group. Scale bar, 50 μm.

The widely accepted method of Annexin V/PI detection using flow cytometry was performed to evaluate the early apoptotic degree (Carew et al., 2006). The ratio of apoptotic RF/6A cells was examined by flow cytometry, which revealed a dramatic increase in the HG group (**Figures 5A, C**; *P* < 0.01). In contrast, Re treatment blocked this increase (**Figures 5A, C**; *P* < 0.01). In addition, TUNEL staining showed that DNA fragmentation was enhanced in HG-treated RF/6A cells, and Re treatment significantly reversed this phenomenon (**Figures 5B, D**; *P* < 0.01). The above findings confirm that Re can protect RF/6A cells from HG-induced apoptosis.

### Ginsenoside Re Inhibited the HIF-1α-Mediated Activation of VEGF in Response to HG

**Figure 6:**
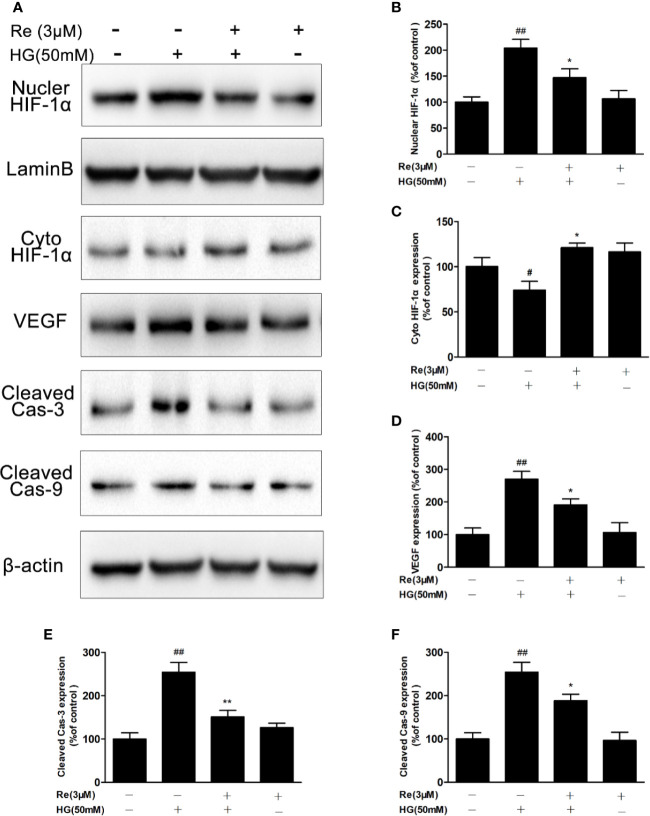
Ginsenoside Re inhibited the HIF-1α-mediated activation of VEGF and apoptosis in response to HG. **(A)** Representative blots of HIF-1α, VEGF and apoptosis-related proteins in RF/6A cells. **(B–F)** Quantitative densitometric analysis of **(B)** nuclear HIF-1α, **(C)** cytoplasmic HIF-1α, **(D)** VEGF, **(E)** cleaved caspase-3, and **(F)** cleaved caspase-9. The results are presented as the mean ± SEM percentage of the control from three independent tests. ^##^*P* < 0.01 versus the control group; *P < 0.05, **P < 0.01 versus the HG group. VEGF, vascular endothelial growth factor.

Based on the above results that ginsenoside Re possesses protective effects and decreases intracellular ROS in HG-induced RF/6A cells, Western blot experiment was conducted to explore whether Re effected the HIF-1α and its downstream correlation pathway VEGF *via* reducing ROS and suppressing apoptosis, resulting in inhibiting malignant proliferation in DR. As shown in **Figures 6A, E, F**, the activated caspase-3 and caspase-9 levels were upregulated by HG treatment (cleaved caspase-3, *P* < 0.01; cleaved caspase-9, *P* < 0.01), and this phenomenon was notably reversed by Re incubation (cleaved caspase-3, *P* < 0.01; cleaved caspase-9, *P* < 0.05).

In addition, the results suggested that HG (50 mM) treatment significantly increased VEGF level (**Figures 6A, D**; *P* < 0.01); however, Re (3 μM) reduced the HG-induced upregulation of VEGF levels (**Figures 6A, D**; *P* < 0.05). HIF-1-α plays a key role in process of oxidative stress, especially the excessive accumulation of ROS, triggering the translocation of genes related to endothelial damage and oxidation. In our study, HG pre-incubation significantly depressed HIF-1α level in the cytoplasm (**Figures 6A, C**; *P* < 0.05) but increased its expression in the nucleus (**Figures 6A, B**; *P* < 0.01). However, Re dramatically decreased the transcription of HIF-1α from the cytoplasm to the nucleus (**Figures 6A–C**; *P* < 0.05), indicating that ginsenoside Re may protect against HG-triggered RF/6A cells injury *via* the HIF-1α/VEGF signal pathway (**Data Sheet 1**).

### Ginsenoside Re Inhibited HIF-1α Signaling Through the PI3K/Akt Pathway

**Figure 7:**
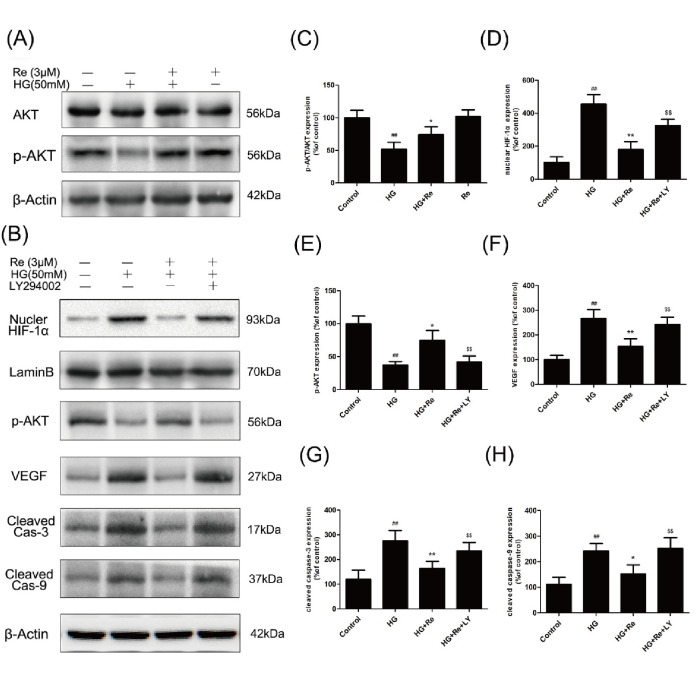
Re protects RF/6A cells *via* regulation of the PI3K/Akt pathway. **(A)** Akt and p-AKT expression detected by Western blot. **(B)**, The changes of related proteins after LY294002 (PI3K inhibitor) incubation. **(C)**, Analysis of Akt and p-Akt expression. **(D–H)** Statistic analysis of related protein levels. The results are presented as the mean ± SEM percentage of the control from three independent tests. ^##^*P* < 0.01 versus the control group; *P < 0.05, **P < 0.01 versus the HG group.^$$^*P* < 0.01 versus the HG + Re group. PI3K, phosphoinositide 3-kinase.

Re enhanced the Akt phosphorylation which was suppressed by HG, as shown in **Figures 7A, C**, suggesting the critical role of Akt signaling pathway.

Subsequently, The LY294002, a PI3K inhibitor, was added to confirm the effect of Akt signaling pathway on the HIF-1α and related apoptotic proteins. Data demonstrated that RF/6A Re exhibited downregulation of cleaved caspase-3 and cleaved caspase-9, which were inhibited by LY294002. Moreover, LY294002 reversed the downregulation of VEGF and nuclear HIF-1α, which was reduced by Re on HG-induced RF/6A cells (**Figures 7B, D–H**). In conclusion, these data (**Data Sheet 1**) indicate that ginsenoside Re mitigated HG-triggered apoptosis mediated by activation of HIF1-α/VEGF in a PI3K/Akt-dependent pattern.

## Discussion

Diabetes is a serious and common chronic metabolic disease, which can result in a variety of diabetes‐related vascular complications or diseases, including diabetic nephropathy, diabetic encephalopathy, and DR (Beckman and Creager, 2016; Zheng et al., 2018). These chronic complications of diabetes are important causes of death and disability, creating in a major public health burden. DR is defined as a serious microvascular complication in diabetics and the major factor of blindness among working-age individuals in most nations (Alswailmi, 2018; Cui et al., 2018; Liu et al., 2018). At present, the mechanisms leading to DR are not fully understood, the general view is that vascular endothelial cell migration and microvascular proliferation caused by VEGF overexpression may be some of the most important mechanisms underlying the development of PDR (Mazidi et al., 2017; Olivares et al., 2017; Shi et al., 2018). Our study reports the impairment of retinal vascular endothelial cells by HG or states mimicking diabetes, and Re pretreatment was shown to exert protective effects against DR through the HIF-1α/VEGF signal in the HG-induced retinal vascular injury model. Hence, The results have profound significance for future research of Re and will increase a certain amount of data of Re as a DR treatment.

In the hyperglycaemic state, aldose reductase activity is increased, and flux through the polyol metabolic pathway can increase up to four times that under normal glucose conditions; consequently, large amounts of sorbitol and fructose accumulate in cells, and the osmotic pressure in retinal tissue changes at the early stage of DR, resulting in neovascularization to reduce hypoxia, which is the first physiological marker of DR (Minamiyama et al., 2010; Costa and Soares, 2013; Jiang et al., 2013; Mei et al., 2018). However, as endothelial cells are exposed to different blood pressure under physiological or pathological conditions, these indicators are difficult to evaluate and therefore are not covered in this study.

On the one hand, tissue hypoxia caused by the decrease in blood flow leads to insufficient energy metabolism by mitochondria, which increases ROS levels and induces the apoptosis of retinal cells in the early environment created by HG (Sifuentes-Franco et al., 2018), which was also proven by our researches. In HG-treated RF/6A cells compared to control cells, intracellular ROS, LDH, and MDA levels were markedly increased, but incubation with Re dramatically suppressed ROS, LDH, and MDA expression and upregulated the antioxidants CAT and GSH-Px level; these results further suggest that Re exhibits its cytoprotective function by reducing oxidative stress and improving mitochondrial function (**Figures 3** and **4**).

On the other hand, hypoxia can significantly increase HIF-1α expression, which induces overexpression of the HIF-1α-associated growth factor VEGF, leading to excessive formation of fibrovascular tissue on the retina and thereby increasing the risk and likelihood of blindness in diabetic patients (Boscia, 2010; Lang, 2012; Rhim et al., 2013; Das et al., 2015; Khodaeian et al., 2015; Mazidi et al., 2017; Tang et al., 2017; Liu et al., 2018). HIF-1α, a hypoxia response protein, is located in the cytoplasm under oxygen-rich conditions and migrates to the nucleus in an anoxic environment, thus promoting angiogenesis in conditions such as DR. The level of HIF-1 and the degree of its nuclear translocation are essential for regulating the oxidation process. It is a complex of two subunits, an oxygen dependent subunit (HIF-1α) and a constitutively expressed nuclear subunit (HIF-1β) (Hagen et al., 2004). Under physiological conditions, HIF-1α expressed in cytoplasm is resolved by 26S proteasome. In hypoxic process, HIF-1α is combined with HIF-1β and remains stable, thereby activating the expression of a series of genes, which are critical for regulating cell metabolism and physiological processes. (Lindenbaum et al., 1972; Cao et al., 2010).

At present, the relationship between HIF-1α and diabetic complications is gradually becoming more recognized. Refer to the past research of HIF-1α in diabetes complications including DR, HIF-1α has become a potential target, and the research of new drugs for treatment of DR will be of great significance for patients suffering from diabetes and its related chronic complications. In our study, how Re mediates the process of HIF-1α and HIF-1β binding is a very interesting point worthy of further study.

**Figure 8:**
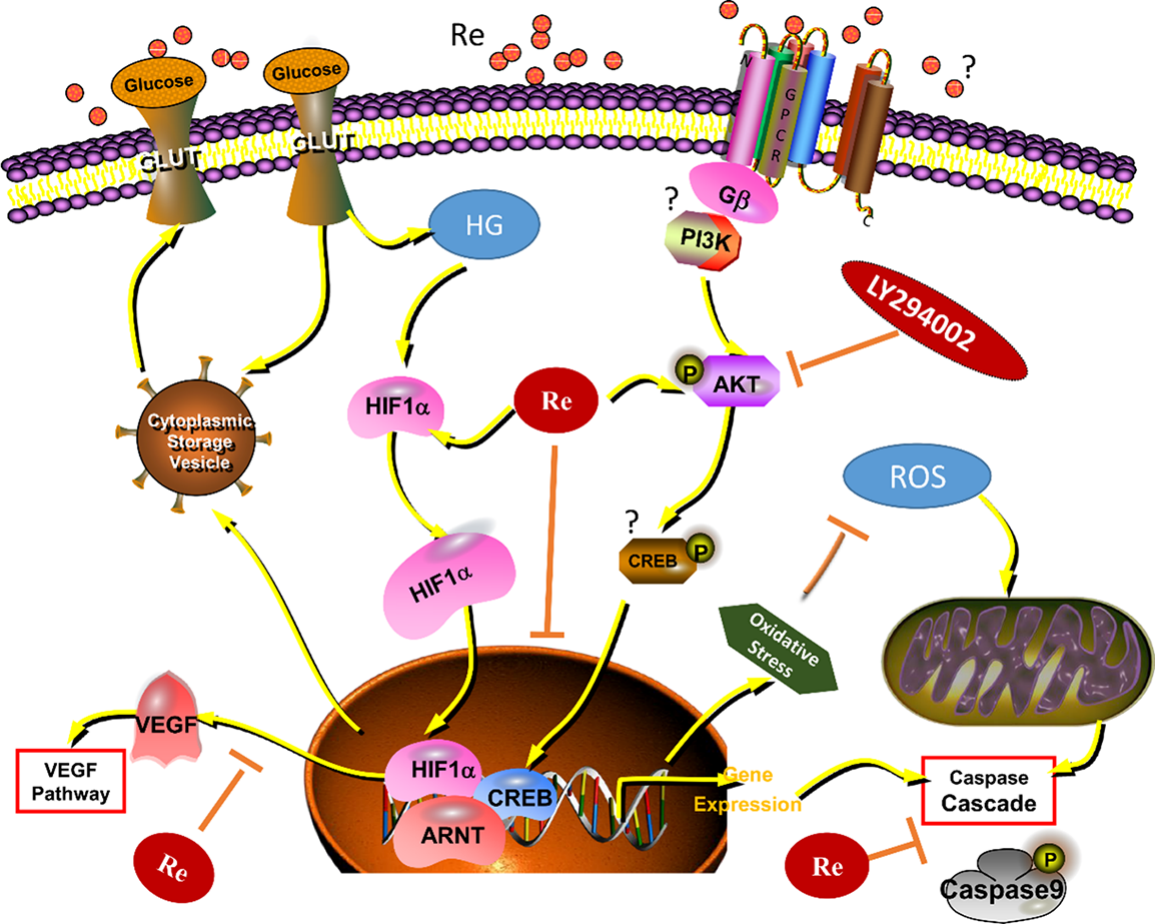
Ginsenoside Re exerts protective effects on retinal microvascular endothelial cells by ameliorating HG-induced retinal angiogenesis and apoptosis *via* the HIF-1α/VEGF signal pathway. It suggests that Ginsenoside Re may reduce cell oxidation injury and mitochondrial apoptosis leaded to by the excessive ROS, regulate the HIF-1α expression and inhibit the activity of VEGF *via* downregulation of oxidative stress and intracellular ROS induced by HG in RF/6A cells, which indicates ginsenoside Re may have an anti-angiogenesis effect in diabetic retinopathy *via* the PI3K/AKT mediated HIF-1α/VEGF signal pathway. HG, high glucose; RE, ginsenoside Re; DR, diabetic retinopathy; “?” means “uncertain.”

Moreover, numerous studies have found that AKT-associated signal is involved in the occurrence and development of DR. In addition, the steady state maintenance of endothelial function is closely related to the activation of PI3K and AKT, which can improve the energy metabolism of endothelial cells and promote cell survival (Huang and Sheibani, 2008). In this study, Re was also found to protect the endothelial cell damage induced by HG is related to the AKT signaling pathway, which was further verified by the treatment of LY294002 (**Figure 8**). Our results indicated that the protective effect of Re on HG-triggered RF/6A cells damage was involved in PI3K/Akt signal regulation.

In conclusion, we found that the natural compound ginsenoside Re showed potential protective activity against DR. Therefore, our research may provide more evidence and basis for DR clinical new drug development. However, as endothelial cells are exposed to different blood pressure under physiological or pathological conditions, their responses largely differ depending on events like shear stress, mechanosensing. The aspects such as mechanosensing cannot be discharged from endothelial readouts. Therefore, these efficacy evaluations need to be further verified clinically.

## Data Availability Statement

The datasets analyzed in this article are publicly available. Requests to access the datasets should be directed to WX, xwjginseng@126.com.

## Author Contributions

PZ, WX, and MQ designed the research. WX, PZ, XZ, and XD performed the experimental work. WX and PZ wrote the manuscript. CZ, ZD, and MQ performed the statistical analysis. GS and XS were responsible for the supervision and project administration. All authors discussed, edited, and approved the ﬁnal version.

## Conflict of Interest

The authors declare that the research was conducted in the absence of any commercial or financial relationships that could be construed as a potential conflict of interest.
