# Corrigendum: Ginsenoside Re Attenuates High Glucose-Induced RF/6A Injury *via* Regulating PI3K/AKT Inhibited HIF-1a/VEGF Signaling Pathway

**DOI:** 10.3389/fphar.2020.01312

**Published:** 2020-10-23

**Authors:** Weijie Xie, Ping Zhou, Muwen Qu, Ziru Dai, Xuelian Zhang, Chenyang Zhang, Xi Dong, Guibo Sun, Xiaobo Sun

**Affiliations:** ^1^Institute of Medicinal Plant Development, Peking Union Medical College and Chinese Academy of Medical Sciences, Beijing, China; ^2^Guang’anmen Hospital, Chinese Academy of Chinese Medical Sciences, Beijing, China

**Keywords:** ginsenoside Re, diabetic retinopathy, oxidative stress, apoptosis, phosphoinositide 3-kinase/AKTT, hypoxia-inducible factor-1-alpha, vascular endothelial growth factor

In the original article, there was a mistake in **Figures 3**, **5** and **7** as published. The marked symbols “+” and “-”in **Figures 3B, C**, **Figures 5C, D** and **Figure 7C** were misplaced. The corrected **Figures 3**, **5** and **7** appear below.

**Figure 3:**
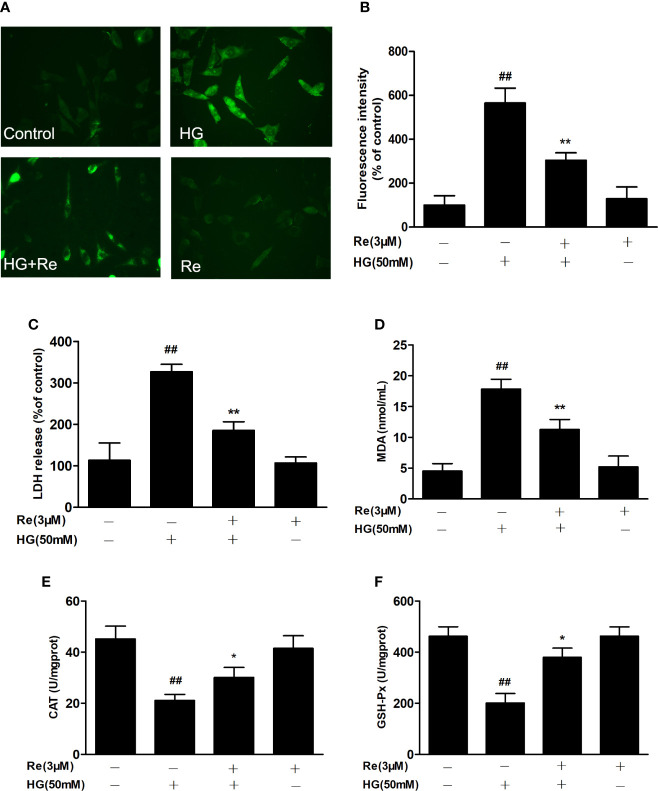
Ginsenoside Re attenuated HG-induced RF/6A cell injury and oxidative stress. **(A)** ROS levels were monitored using a fluorescence microscope. **(B)** Statistical analysis of ROS fluorescence intensity. The enzymatic activities of LDH **(C)**, MDA **(D)**, CAT **(E)**, and GSH-Px **(F)** were detected by spectrophotometry. The data are presented as the mean ± standard error of the mean (n = 5). ^##^*P* < 0.01 versus the control group; *P < 0.05, **P < 0.01 versus the HG group. Scale bar, 50 μm.

**Figure 5:**
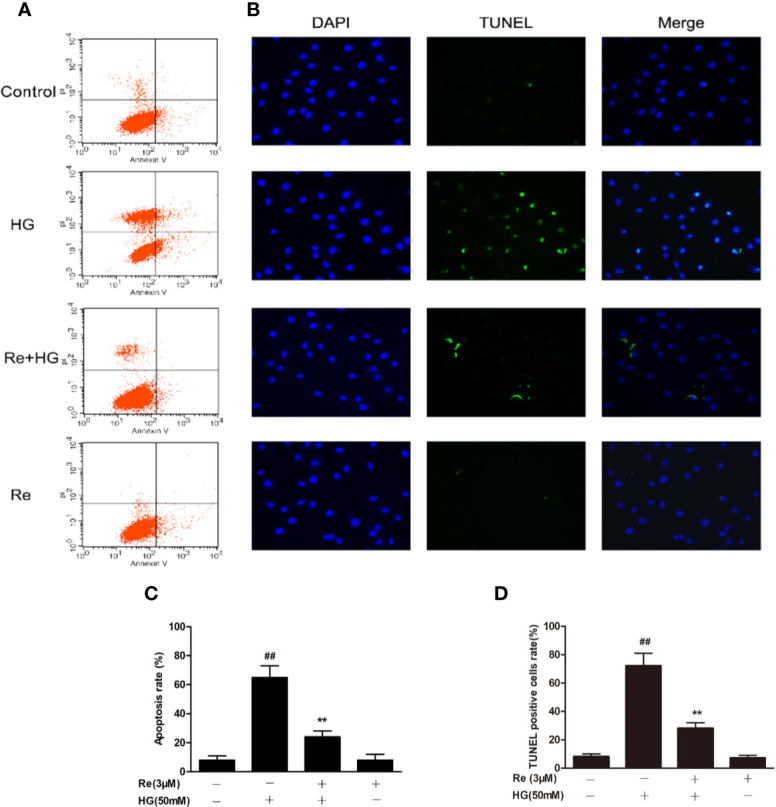
Effects of Ginsenoside Re on HG-triggered apoptosis in RF/6A cells. **(A)** Distribution map of apoptotic cells detected by annexin V/PI double staining. **(B)** Representative images captured with fluorescence microscopy showing TUNEL-stained RF/6A cells. **(C)** Quantitative analysis of the ratio of annexin V/PI-positive cells to total cells. **(D)** The ratio of TUNEL-positive cells. The results are expressed as the mean ± SE of the mean (n = 5). ^##^*P* < 0.01 versus the control group; **P < 0.01 versus the HG group. Scale bar, 50 μm.

**Figure 7:**
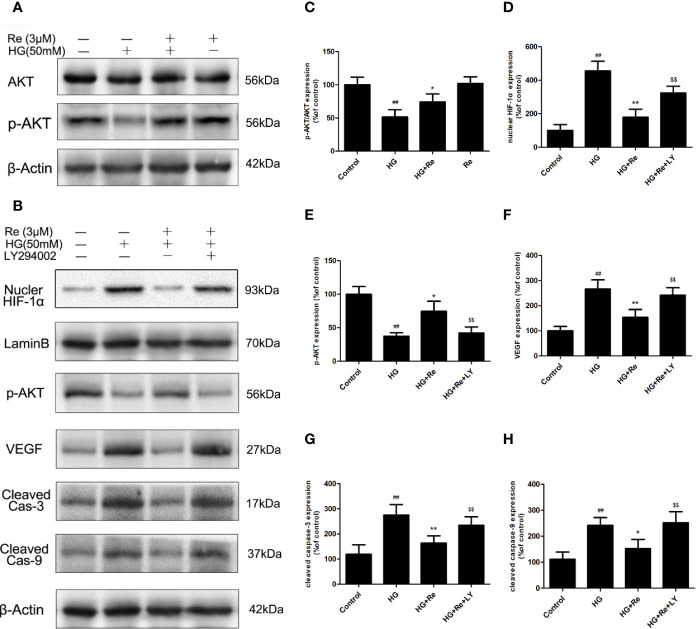
Re protects RF/6A cells *via* regulation of the PI3K/Akt pathway. **(A)** Akt and p-AKT expression detected by western blot. **(B)** The changes of related proteins after LY294002 (PI3K inhibitor) incubation. **(C)** Analysis of Akt and p-Akt expression. **(D**–**H)** Statistic analysis of related protein levels. The results are presented as the mean ± SEM percentage of the control from three independent tests. ^##^*P* < 0.01 versus the control group; *P < 0.05, **P < 0.01 versus the HG group; ^$$^*P* < 0.01 versus the HG+Re group.

The authors apologize for this error and state that this does not change the scientific conclusions of the article in any way. The original article has been updated.

