# Corrigendum: Ginsenoside Re attenuates high glucose-induced RF/6A injury via regulating PI3K/AKT inhibited HIF-1a/VEGF signaling pathway

**DOI:** 10.3389/fphar.2024.1451696

**Published:** 2024-07-19

**Authors:** Weijie Xie, Ping Zhou, Muwen Qu, Ziru Dai, Xuelian Zhang, Chenyang Zhang, Xi Dong, Guibo Sun, Xiaobo Sun

**Affiliations:** ^1^ Institute of Medicinal Plant Development, Peking Union Medical College and Chinese Academy of Medical Sciences, Beijing, China; ^2^ Guang’anmen Hospital, Chinese Academy of Chinese Medical Sciences, Beijing, China

**Keywords:** ginsenoside Re, diabetic retinopathy, oxidative stress, apoptosis, phosphoinositide 3-kinase/AKTT, hypoxia-inducible factor-1-alpha, vascular endothelial growth factor

In the original article, there was a mistake in the Figure 7 as published. The protein band of β-actin in Figure 7B were misplaced**.** The corrected Figure 7 and its caption appears below.

**FIGURE 7:**
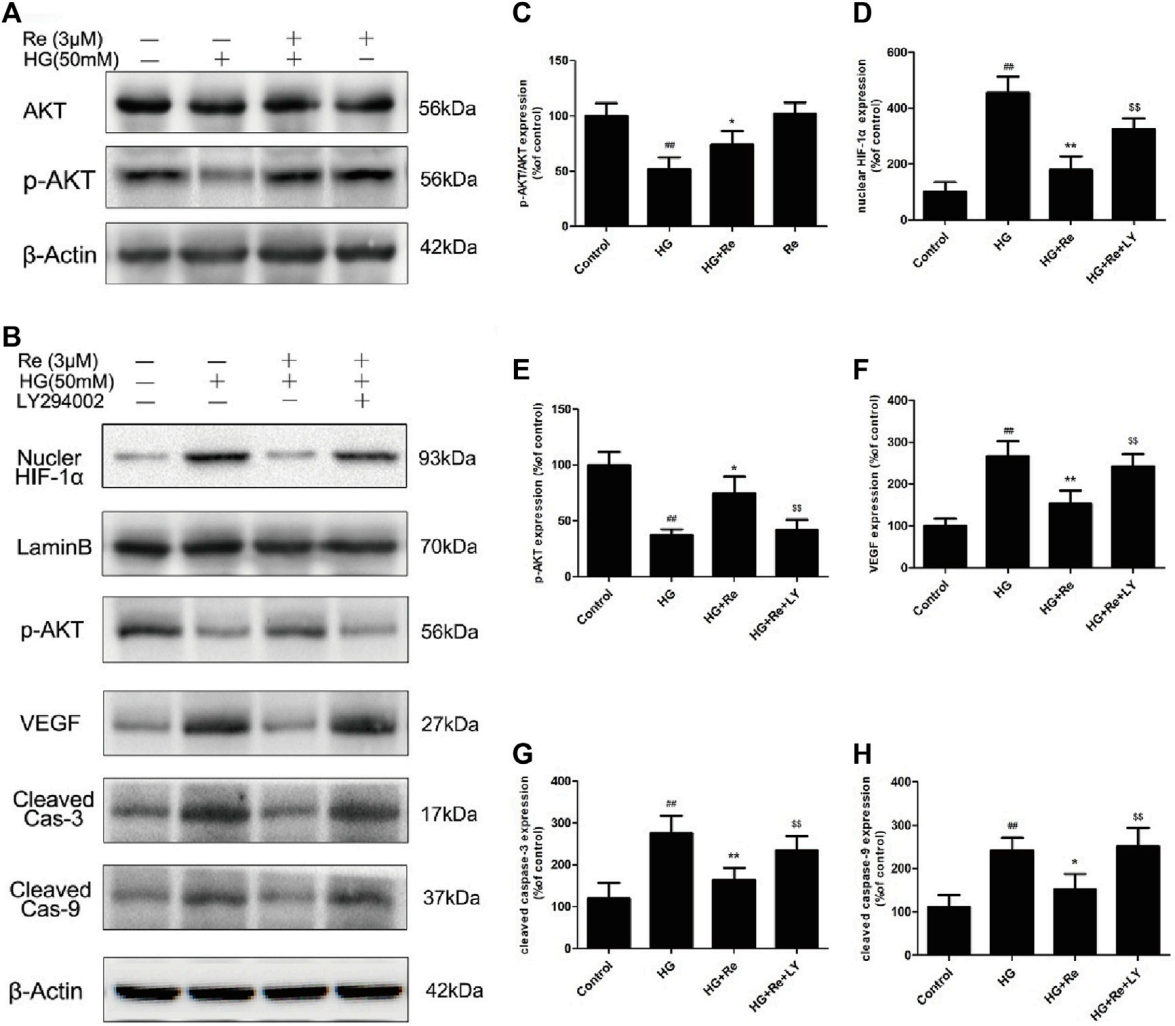
Re protects RF/6A cells via regulation of the PI3K/Akt pathway. **(A)**, Akt and p-AKT expression detected by western blot. **(B)**, The changes of related proteins after LY294002 (PI3K inhibitor) incubation. **(C)**, Analysis of Akt and p-Akt expression. **(D–H)**, Statistic analysis of related protein levels. The results are presented as the mean ± SEM percentage of the control from three independent tests. ##*p* < 0.01 *versus* the control group; *p < 0.05, **p < 0.01 *versus* the HG group.

The authors apologize for this error and state that this does not change the scientific conclusions of the article in any way. The original article has been updated.

