# Supplementary figures and images for: Ginsenoside Re Attenuates High Glucose-Induced RF/6A Injury via Regulating PI3K/AKT Inhibited HIF-1α/VEGF Signaling Pathway
Source: Front Pharmacol. 2020 May 21;11:695. doi: 10.3389/fphar.2020.00695 (PMC7253708; doi:10.3389/fphar.2020.00695)

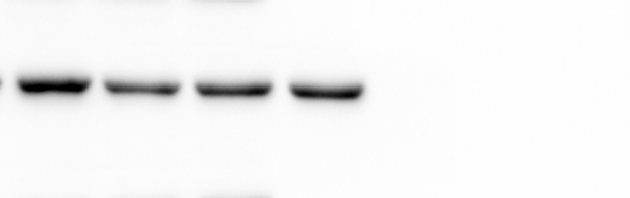

Supplement: Supplementary file 1 [file DataSheet_1.zip › protein expression/Fig.6A/Cyto HIF-1a/╡┌╥╗┤╬/Cyto HIF-1a.tif]

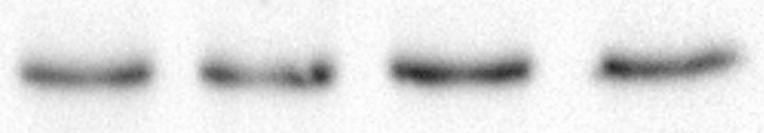

Supplement: Supplementary file 1 [file DataSheet_1.zip › protein expression/Fig.6A/Cyto HIF-1a/╡┌╚2┤╬/cyto-HIF-1a┴.tif]

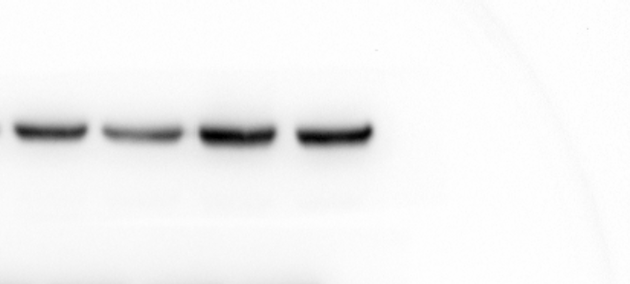

Supplement: Supplementary file 1 [file DataSheet_1.zip › protein expression/Fig.6A/Cyto HIF-1a/╡┌╢■┤╬/Cyto HIF-1a.tif]

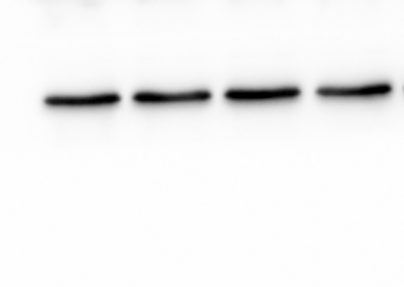

Supplement: Supplementary file 1 [file DataSheet_1.zip › protein expression/Fig.6A/Lamin B/╡┌╥╗┤╬/laminB.tif]

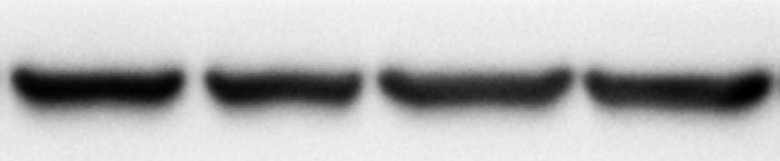

Supplement: Supplementary file 1 [file DataSheet_1.zip › protein expression/Fig.6A/Lamin B/╡┌╚2┤╬/laminB.tif]

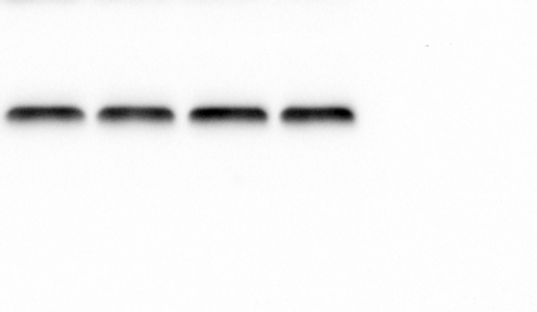

Supplement: Supplementary file 1 [file DataSheet_1.zip › protein expression/Fig.6A/Lamin B/╡┌╢■┤╬/LaminB.tif]

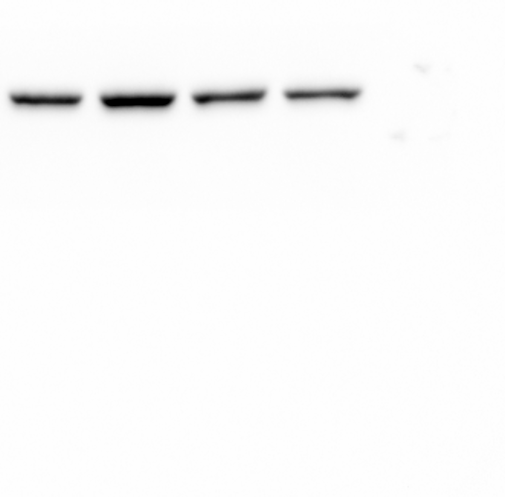

Supplement: Supplementary file 1 [file DataSheet_1.zip › protein expression/Fig.6A/N HIF-1a/╡┌╥╗┤╬/Nuclear HIF1.tif]

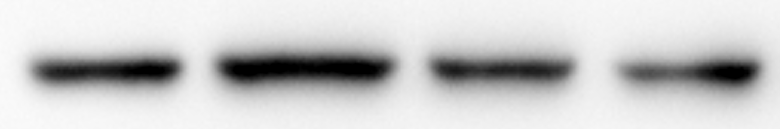

Supplement: Supplementary file 1 [file DataSheet_1.zip › protein expression/Fig.6A/N HIF-1a/╡┌╚2┤╬/nucler HIF-1.tif]

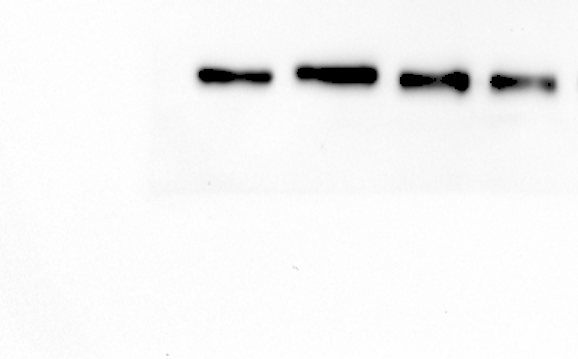

Supplement: Supplementary file 1 [file DataSheet_1.zip › protein expression/Fig.6A/N HIF-1a/╡┌╢■┤╬/HIF1.tif]

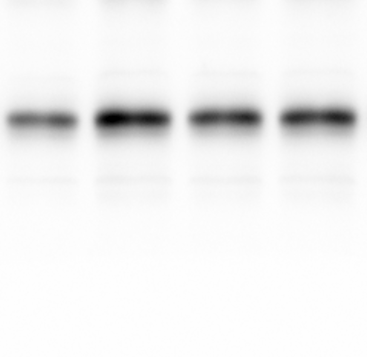

Supplement: Supplementary file 1 [file DataSheet_1.zip › protein expression/Fig.6A/VEGF/╡┌╥╗┤╬/VEGF.tif]

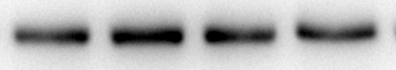

Supplement: Supplementary file 1 [file DataSheet_1.zip › protein expression/Fig.6A/VEGF/╡┌╚2┤╬/VEGF.tif]

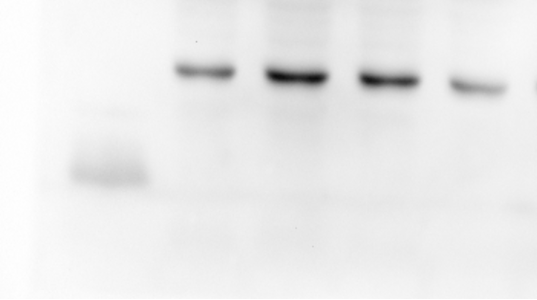

Supplement: Supplementary file 1 [file DataSheet_1.zip › protein expression/Fig.6A/VEGF/╡┌╢■┤╬/VEGF.tif]

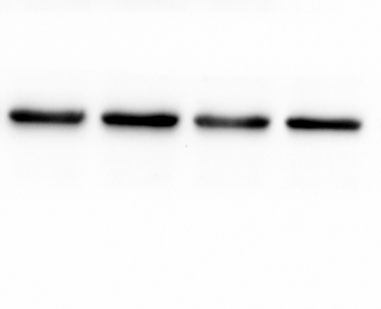

Supplement: Supplementary file 1 [file DataSheet_1.zip › protein expression/Fig.6A/actin/first/actin.tif]

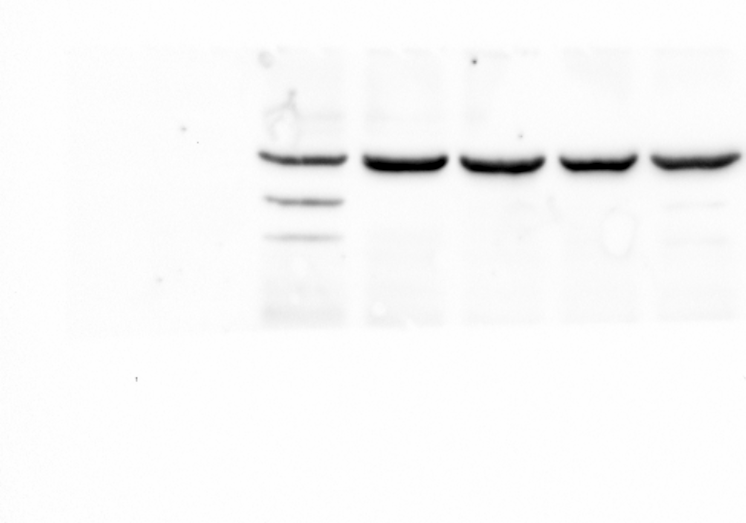

Supplement: Supplementary file 1 [file DataSheet_1.zip › protein expression/Fig.6A/actin/second/actin.tif]

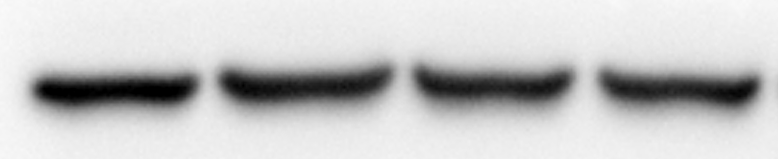

Supplement: Supplementary file 1 [file DataSheet_1.zip › protein expression/Fig.6A/actin/third/actin.tif]

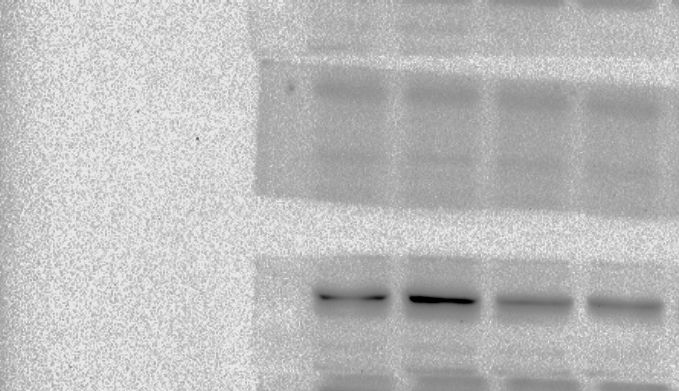

Supplement: Supplementary file 1 [file DataSheet_1.zip › protein expression/Fig.6A/cas-3/first/caspase-3-1.tif]

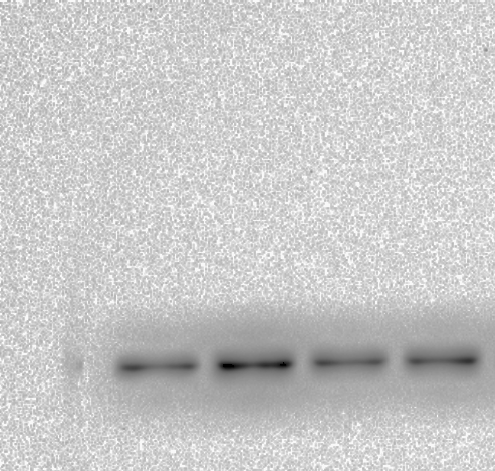

Supplement: Supplementary file 1 [file DataSheet_1.zip › protein expression/Fig.6A/cas-3/second/cas-3.tif]

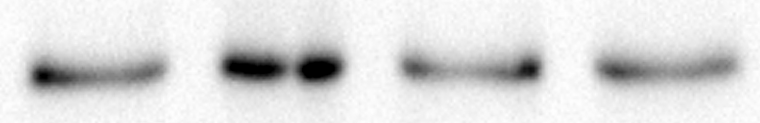

Supplement: Supplementary file 1 [file DataSheet_1.zip › protein expression/Fig.6A/cas-3/third/cas-3.tif]

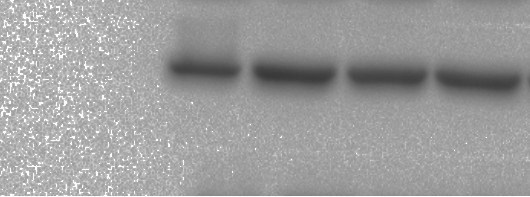

Supplement: Supplementary file 1 [file DataSheet_1.zip › protein expression/Fig.6A/cas-9/first/cas-9.tif]

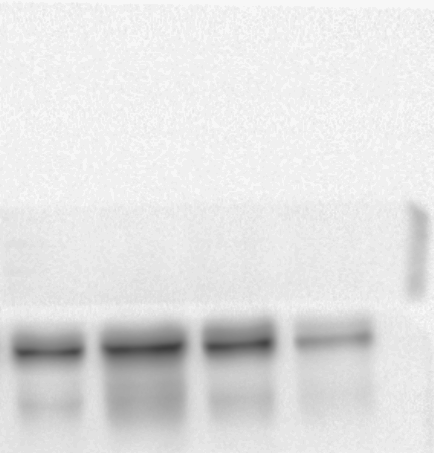

Supplement: Supplementary file 1 [file DataSheet_1.zip › protein expression/Fig.6A/cas-9/second/cas-9.tif]

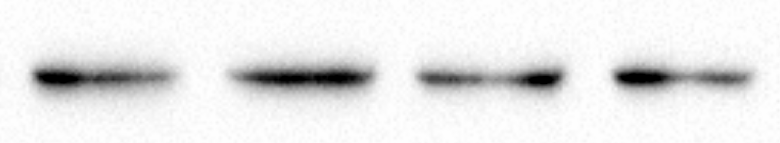

Supplement: Supplementary file 1 [file DataSheet_1.zip › protein expression/Fig.6A/cas-9/third/cc-9.tif]

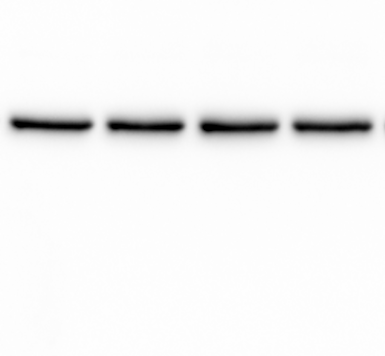

Supplement: Supplementary file 1 [file DataSheet_1.zip › protein expression/Fig.7/Fig.7A/Akt/╡┌╥╗┤╬/AKT.tif]

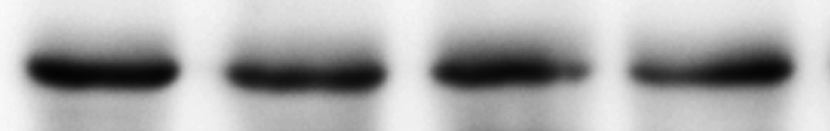

Supplement: Supplementary file 1 [file DataSheet_1.zip › protein expression/Fig.7/Fig.7A/Akt/╡┌╚2┤╬/AKT.tif]

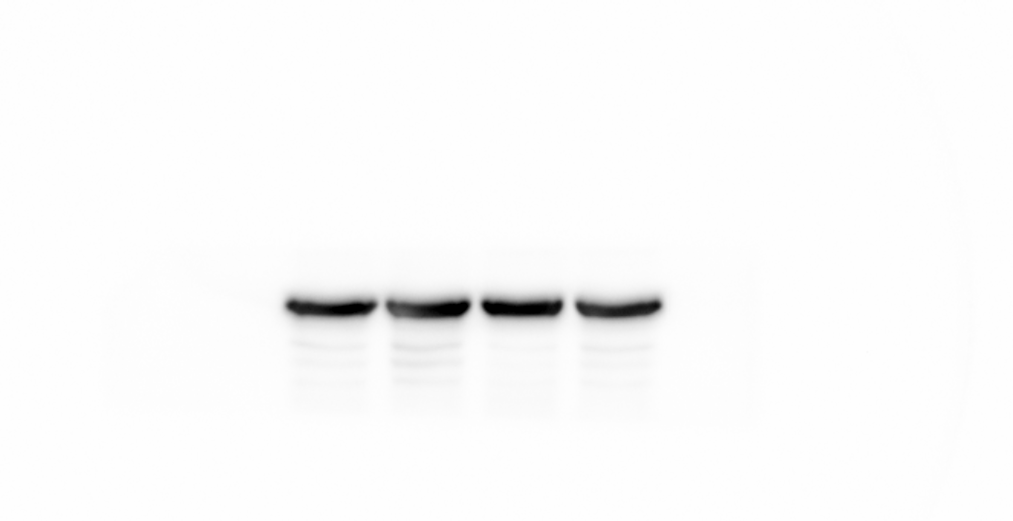

Supplement: Supplementary file 1 [file DataSheet_1.zip › protein expression/Fig.7/Fig.7A/Akt/╡┌╢■┤╬/Akt.tif]

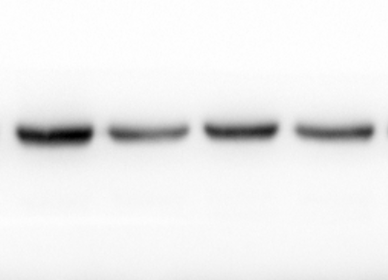

Supplement: Supplementary file 1 [file DataSheet_1.zip › protein expression/Fig.7/Fig.7A/P-Akt/╡┌╥╗┤╬/p-Akt.tif]

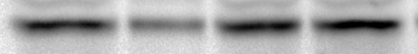

Supplement: Supplementary file 1 [file DataSheet_1.zip › protein expression/Fig.7/Fig.7A/P-Akt/╡┌╚2┤╬/pAKT.tif]

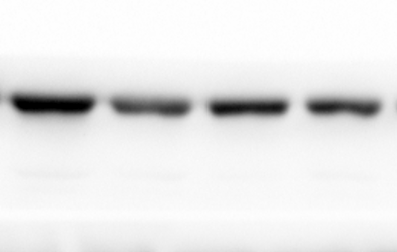

Supplement: Supplementary file 1 [file DataSheet_1.zip › protein expression/Fig.7/Fig.7A/P-Akt/╡┌╢■┤╬/p-Akt.tif]

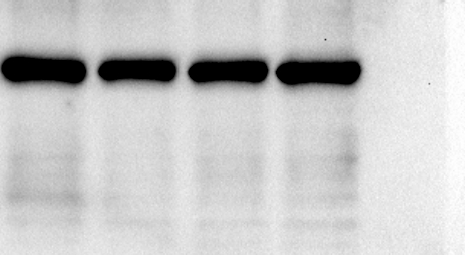

Supplement: Supplementary file 1 [file DataSheet_1.zip › protein expression/Fig.7/Fig.7A/actin/╡┌╥╗┤╬/Administrator 2018-09-30 14 ╨í╩▒ 51 ╖╓╓╙_Exposure_38.3sec.tif]

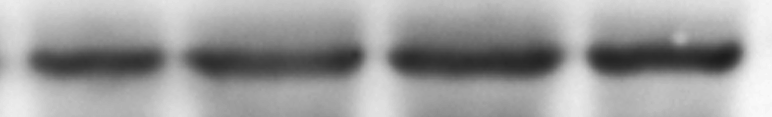

Supplement: Supplementary file 1 [file DataSheet_1.zip › protein expression/Fig.7/Fig.7A/actin/╡┌╚2┤╬/actin.tif]

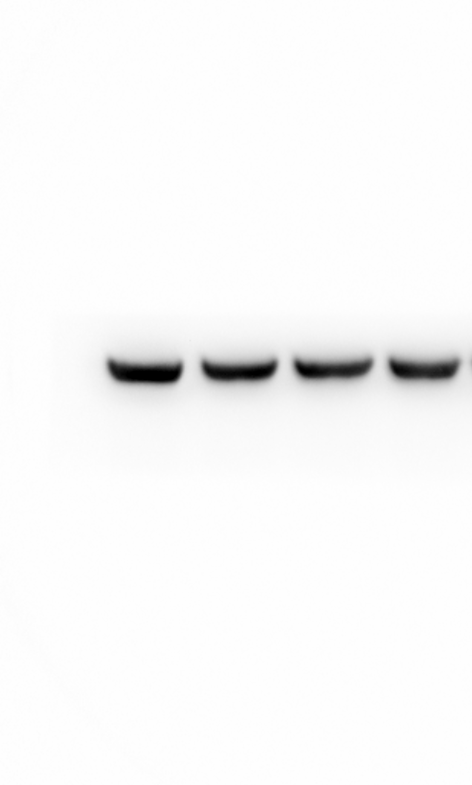

Supplement: Supplementary file 1 [file DataSheet_1.zip › protein expression/Fig.7/Fig.7A/actin/╡┌╢■┤╬/actin.tif]

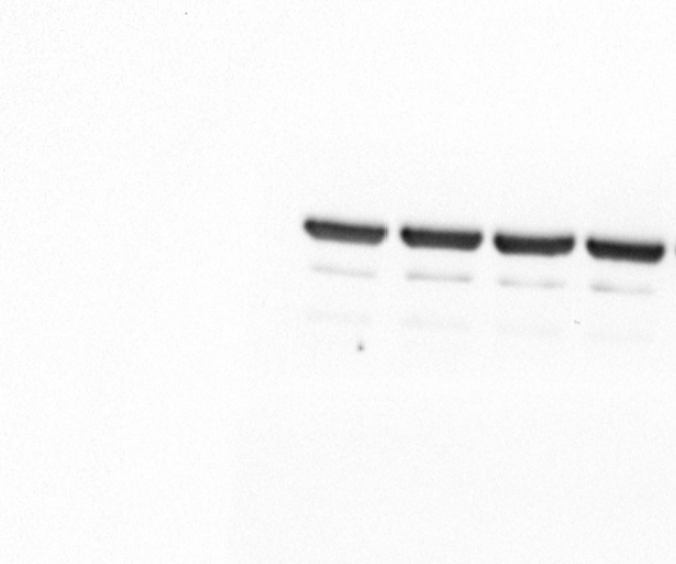

Supplement: Supplementary file 1 [file DataSheet_1.zip › protein expression/Fig.7/Fig.7C/Akt/╡┌╥╗┤╬/Akt.tif]

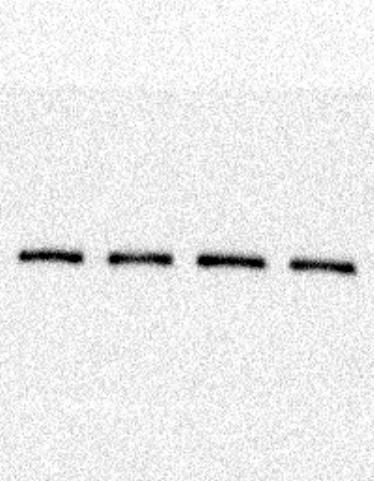

Supplement: Supplementary file 1 [file DataSheet_1.zip › protein expression/Fig.7/Fig.7C/Akt/╡┌╢■┤╬/Akt.tif]

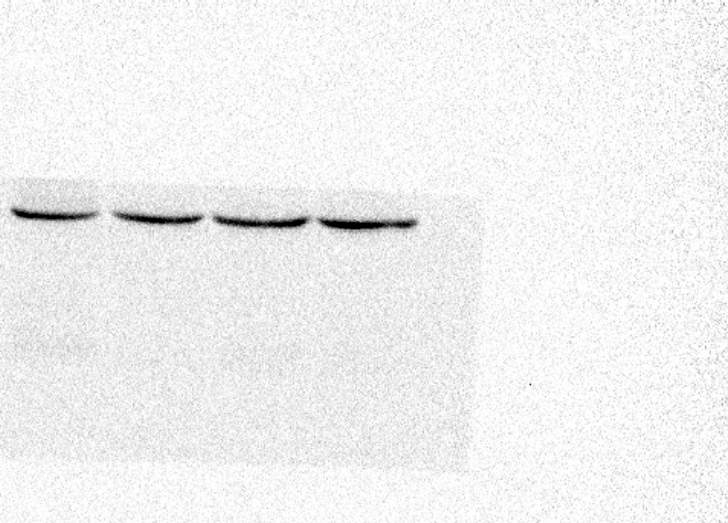

Supplement: Supplementary file 1 [file DataSheet_1.zip › protein expression/Fig.7/Fig.7C/Lamin B/╡┌╥╗┤╬/Lamin B.tif]

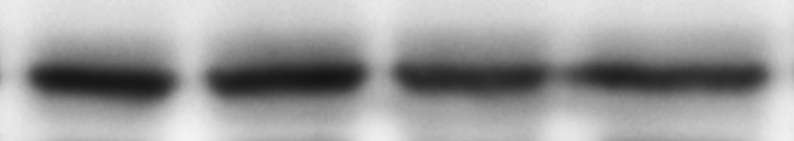

Supplement: Supplementary file 1 [file DataSheet_1.zip › protein expression/Fig.7/Fig.7C/Lamin B/╡┌╚2┤╬/LaminB.tif]

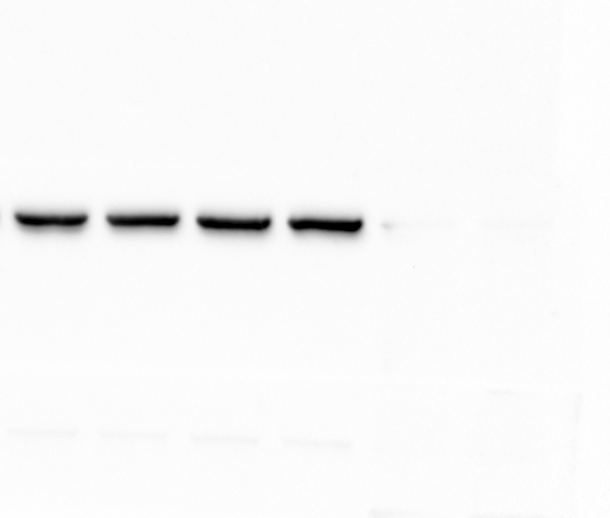

Supplement: Supplementary file 1 [file DataSheet_1.zip › protein expression/Fig.7/Fig.7C/Lamin B/╡┌╢■┤╬/lamin B.tif]

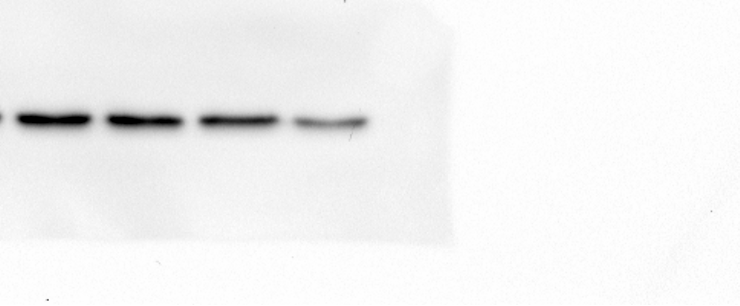

Supplement: Supplementary file 1 [file DataSheet_1.zip › protein expression/Fig.7/Fig.7C/Nuclear HIF-1/╡┌╥╗┤╬/Nuclear HIF-1.tif]

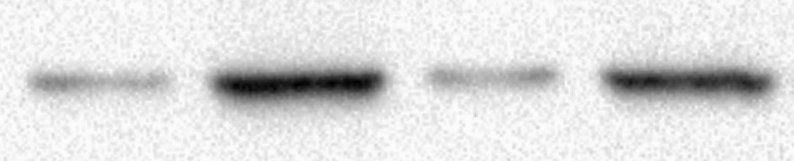

Supplement: Supplementary file 1 [file DataSheet_1.zip › protein expression/Fig.7/Fig.7C/Nuclear HIF-1/╡┌╚2┤╬/HIF-1a,NC.tif]

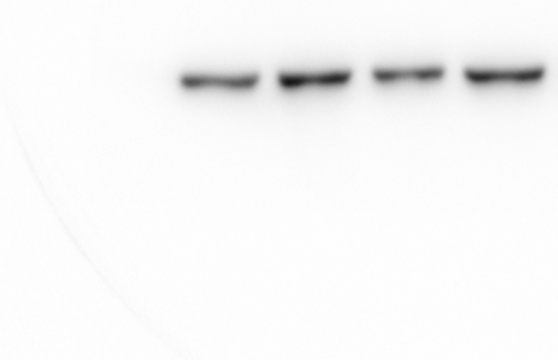

Supplement: Supplementary file 1 [file DataSheet_1.zip › protein expression/Fig.7/Fig.7C/Nuclear HIF-1/╡┌╢■┤╬/Nuclear HIF-1.tif]

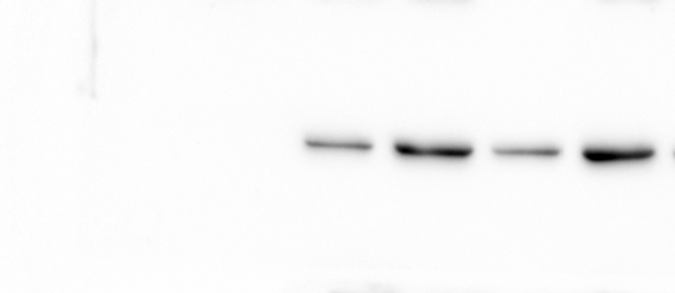

Supplement: Supplementary file 1 [file DataSheet_1.zip › protein expression/Fig.7/Fig.7C/VEGF/╡┌╥╗┤╬/VEGF.tif]

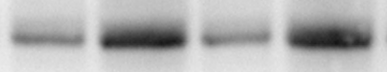

Supplement: Supplementary file 1 [file DataSheet_1.zip › protein expression/Fig.7/Fig.7C/VEGF/╡┌╚2┤╬/VEGF.tif]

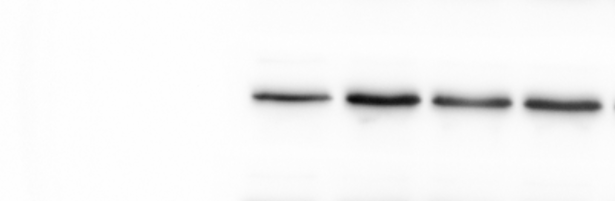

Supplement: Supplementary file 1 [file DataSheet_1.zip › protein expression/Fig.7/Fig.7C/VEGF/╡┌╢■┤╬/VEGF.tif]

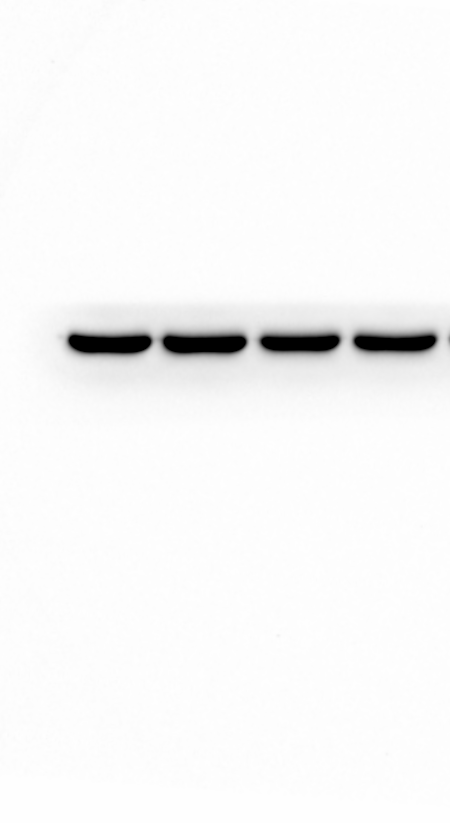

Supplement: Supplementary file 1 [file DataSheet_1.zip › protein expression/Fig.7/Fig.7C/actin/╡┌╥╗┤╬/Administrator 2016-01-17 17 ╨í╩▒ 49 ╖╓╓╙_Exposure_62.4sec.tif]

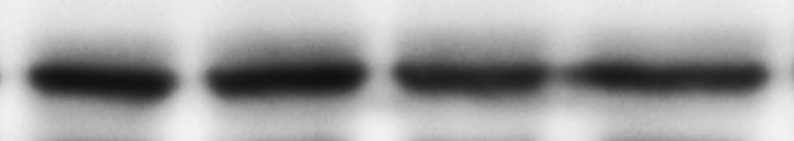

Supplement: Supplementary file 1 [file DataSheet_1.zip › protein expression/Fig.7/Fig.7C/actin/╡┌╚2┤╬/actin.tif]

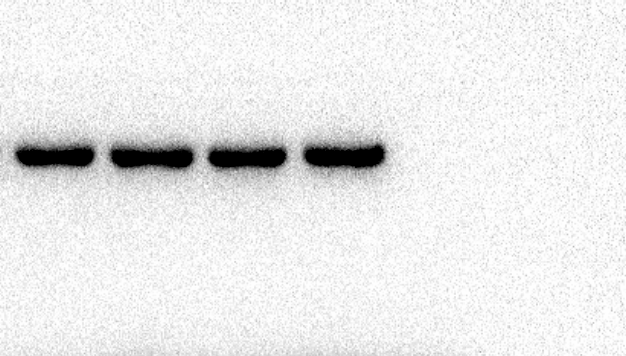

Supplement: Supplementary file 1 [file DataSheet_1.zip › protein expression/Fig.7/Fig.7C/actin/╡┌╢■┤╬/actin.tif]

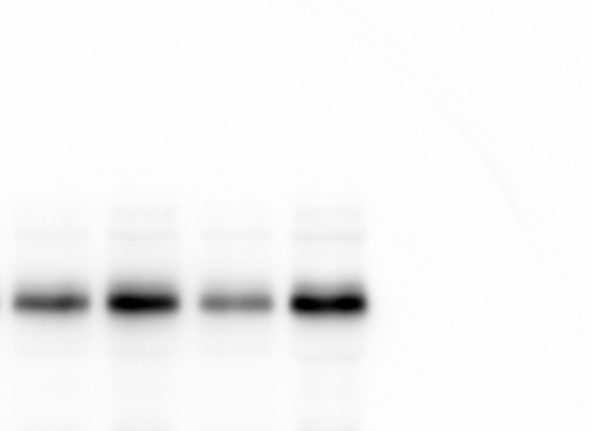

Supplement: Supplementary file 1 [file DataSheet_1.zip › protein expression/Fig.7/Fig.7C/cas-3/╡┌╥╗┤╬/caspase-3.tif]

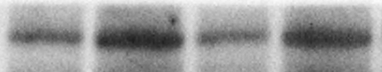

Supplement: Supplementary file 1 [file DataSheet_1.zip › protein expression/Fig.7/Fig.7C/cas-3/╡┌╚2┤╬/c-Cas-3.tif]

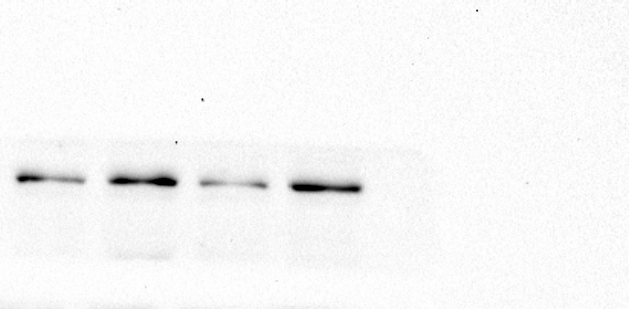

Supplement: Supplementary file 1 [file DataSheet_1.zip › protein expression/Fig.7/Fig.7C/cas-3/╡┌╢■┤╬/caspase-3.tif]

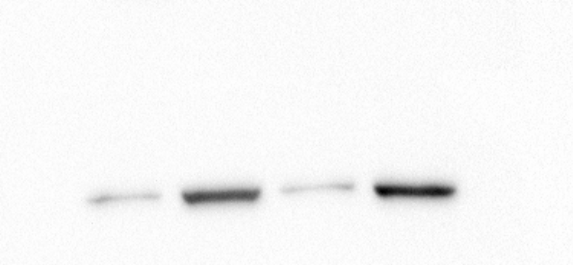

Supplement: Supplementary file 1 [file DataSheet_1.zip › protein expression/Fig.7/Fig.7C/cas-9/╡┌╥╗┤╬/caspase-9.tif]

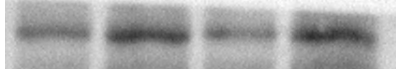

Supplement: Supplementary file 1 [file DataSheet_1.zip › protein expression/Fig.7/Fig.7C/cas-9/╡┌╚2┤╬/c-Cas-9.tif]

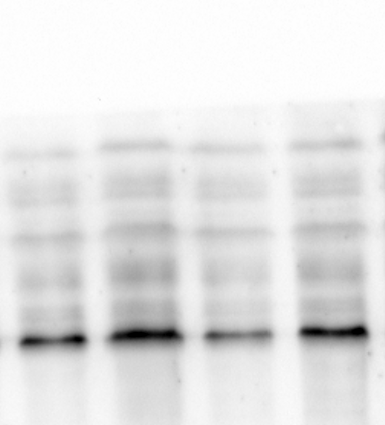

Supplement: Supplementary file 1 [file DataSheet_1.zip › protein expression/Fig.7/Fig.7C/cas-9/╡┌╢■┤╬/caspase-9.tif]

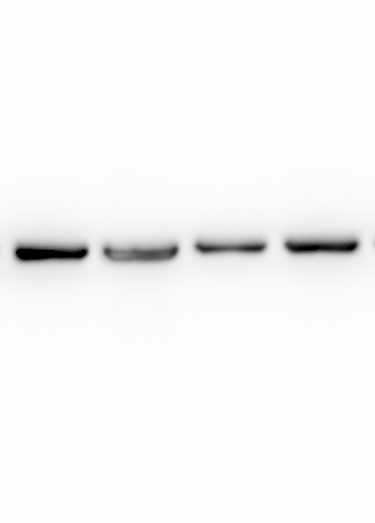

Supplement: Supplementary file 1 [file DataSheet_1.zip › protein expression/Fig.7/Fig.7C/p-Akt/╡┌╥╗┤╬/p-Akt.tif]

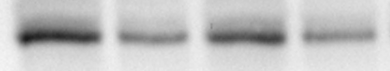

Supplement: Supplementary file 1 [file DataSheet_1.zip › protein expression/Fig.7/Fig.7C/p-Akt/╡┌╚2┤╬/p-AKT.tif]

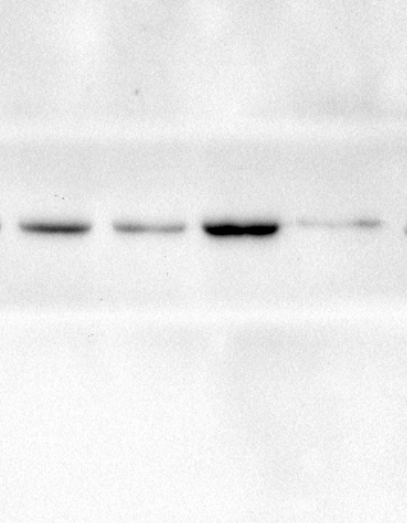

Supplement: Supplementary file 1 [file DataSheet_1.zip › protein expression/Fig.7/Fig.7C/p-Akt/╡┌╢■┤╬/p-Akt.tif]

The graphical view of the experimental design


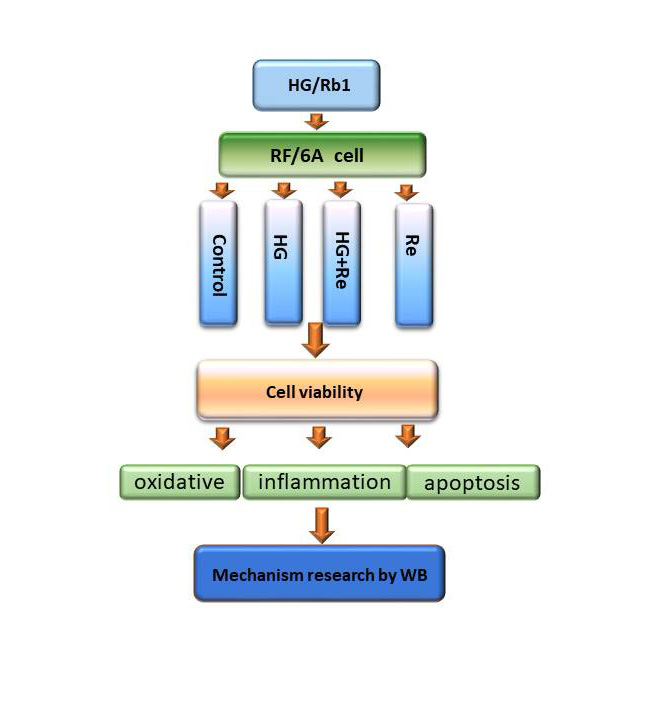

Supplement: Supplementary file 3 [file Table_2.docx]
