# Supplementary material for: Ginsenoside Re Attenuates High Glucose-Induced RF/6A Injury via Regulating PI3K/AKT Inhibited HIF-1α/VEGF Signaling Pathway
Source: Front Pharmacol. 2020 May 21;11:695. doi: 10.3389/fphar.2020.00695 (PMC7253708; doi:10.3389/fphar.2020.00695)

**Supplementary materials**


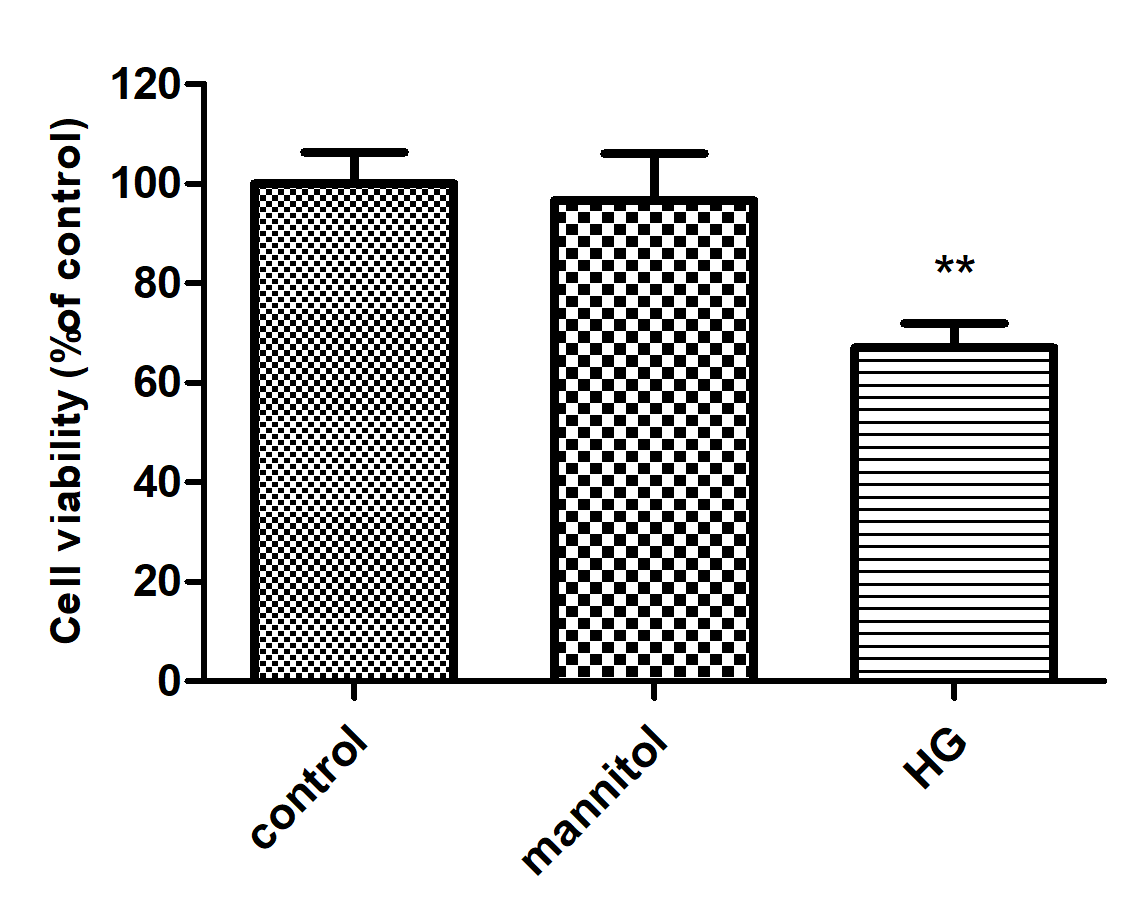


**Figure S1. 50mM HG preconditioning exerted no** **osmotic toxicity on RF/6A cells**

Pro caspase 3 Pro caspase 3 Pro caspase 3


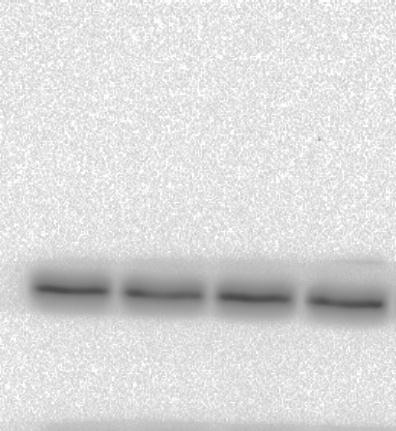

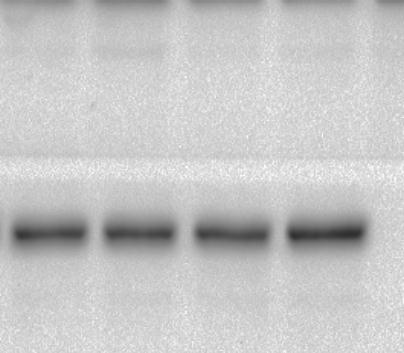

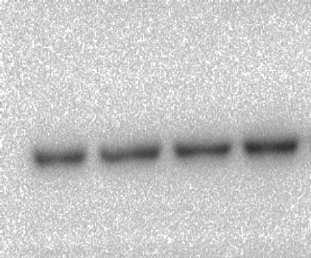

Supplement: Supplementary file 2 [file Table_1.docx]
